# Dietary steviol glycosides mixture supplementation modulates the gene expression of gut chemoreceptors and enhances the antioxidant capacity in weaned piglets

**DOI:** 10.1186/s40813-024-00414-5

**Published:** 2025-02-06

**Authors:** Yunxia Xiong, Zhentao He, Qiwen Wu, Hao Xiao, Shuting Cao, Xuefen Yang, Yajing Li, Zongyong Jiang, Cui Zhu, Li Wang

**Affiliations:** 1 State Key Laboratory of Swine and Poultry Breeding Industry, Key Laboratory of Animal Nutrition and Feed Science in South China Ministry of Agriculture, Heyuan Branch,Guangdong Laboratory for Lingnan Modern Agriculture, Guangdong Key Laboratory of Animal Breeding and Nutrition, Institute of Animal Science, Guangdong Academy of Agricultural Sciences, Guangzhou, Guangdong 510640 China; 2 School of Animal Science and Technology, Foshan University, Foshan, 528225 China; 3 Dongtai Hirye Biotechnology Co., Ltd, Dongtai, 224200 China

**Keywords:** Steviol glycosides, Gut chemoreceptors, Antioxidant capacity, Weaned piglets

## Abstract

**Background:**

Stevia glycosides (SGs) have been widely used as an ideal sugar alternative in the food industry. However, the potential application of SGs mixture in the diets of weaned piglets remains unexplored. This study aimed to investigate the effect of dietary SGs mixture supplementation on growth performance, gene expression of gut chemoreceptors, and antioxidant capacity in weaned piglets.

**Methods:**

A total of 216 weaned piglets (Duroc × Landrace × Yorkshire, 7.36 ± 0.04 kg body weight) were randomly assigned to 6 groups (6 pens/group with 6 piglets/pen), and were fed with the basal diet supplemented with 0, 100, 150, 200, 250, or 300 mg/kg SGs mixture for 42 days. The serum, liver, *longissimus thoracis*, and jejunal samples were collected on day 43.

**Results:**

The results showed that inclusion the SGs mixture in the diet did not have a significant impact on growth performance from days 1 to 28 (*P* > 0.05). But increasing the concentration of SGs mixture tended to linearly decrease the average daily gain from days 1 to 42 (*P* = 0.052). However, 150 mg/kg SGs mixture supplementation significantly increased the mRNA expression of taste receptor family 1 member 2 (*T1R2*) and glucose transporters 2 (*GLUT2*) in the jejunum (*P* < 0.05), while 150 and 200 mg/kg SGs mixture supplementation significantly increased T1R3 mRNA expression (*P* < 0.05). Moreover, 150 mg/kg SGs mixture supplementation significantly reduced serum malondialdehyde content (*P* < 0.05). Increasing the concentration of SGs mixture linearly and quadratically increased serum total superoxide dismutase (T-SOD), catalase (CAT), and glutathione peroxidase (GSH-Px) activity, as well as hepatic T-SOD, GSH-Px activity, and muscle total antioxidant capacity contents (*P* < 0.05). Furthermore, piglets fed a diet supplemented with 100 mg/kg SGs mixture had higher serum T-SOD, CAT, and GSH-Px activities compared with the other treatments (*P* < 0.05).

**Conclusions:**

Therefore, our results suggest that dietary 100 ~ 150 mg/kg SGs mixture supplementation modulates gene expression of sweet taste recognition receptors and glucose transporters, while also enhancing the antioxidant capacity of weaned piglets.

## Background

Steviol glycosides (SGs), a group of diterpenoid glycosides derived from *Stevia rebaudiana* that are 200 ~ 300 times sweeter than sucrose, have become an ideal sugar alternative [1]. To date, 64 SGs have been identified in the leaves of *Stevia rebaudiana*. Among these, ten glycosides, including Stevioside, Rebaudioside A, Rebaudioside B, Rebaudioside C, Rebaudioside D, Rebaudioside E, Rebaudioside F, Dulcoside A, Rubusoside, and Steviolbioside, are found in relatively high abundance [2]. The primary differences in the chemical structures of these SGs lie in the R1 and R2 groups, which are attached at the C13-hydroxyl and C19-carboxyl positions, respectively [2]. SGs are known for their heat stability, pH stability, non-caloric properties, and non-fermentative nature [3]. Over the past decades, SGs have been demonstrated to be non-toxic, devoid of side effects, non-carcinogenic, and safe for consumption, leading to their widespread use in the food and pharmaceutical industries [4, 5]. Beyond sweetness, SGs has multiple bioactivities such as anti-diabetes, anti-hypertension, anti-oxidation, anti-inflammation, anti-microbial, anti-cancer, and anti-diarrheal [3, 6–9], with potential performance benefits for livestock and poultry.

Weaning stress frequently leads to a decrease in feed intake in weaned piglets. The addition of sweeteners such as sucrose, glucose, lactose, and natural or artificial sweeteners to diets improves palatability and helps piglets cope with the reduced appetite that results from weaning stress [10–13]. Previous studies have shown that SGs have the ability to increase feed intake in broiler chickens and goats [14–16]. Wang et al. [17] found that increasing the dietary stevioside/rebaudioside A supplementation from 0 to 300 mg/kg led to a linear increase in average daily feed intake in weaned piglets. However, another study showed that dietary stevia addition had no beneficial effect on feed intake in newly weaned piglets [18]. The steviol glycosides products used in previous studies were nearly pure, exceeding 90%, and it is well-known that purifying products in the industry is a costly process. We hypothesize whether replacing these purified products with a SGs mixture, which is a more affordable option with a simpler production process, would positively impact food intake in weaned piglets. Moreover, in mammals, sweet molecules are typically detected by chemoreceptors, which then stimulate pathways associated with appetite and feed intake [19]. The role gut chemoreceptors play in SGs mixture sensing needs further research. On the other hand, weaning stress can induce the body to produce much free radicals, resulting in oxidative stress [20]. Besides, stevioside has been widely reported to have excellent antioxidant effect, which can alleviate the damage induced by oxidative stress [21]. Whether the SGs mixture could alleviate the oxidative stress in weaned piglets is still unclear. Therefore, this study was conducted to investigate the effects of dietary SGs mixture supplementation on performance, expression of gut chemoreceptors, and antioxidant capacity.

## Methods

### Steviol glycosides mixture compositions

**Fig. 1:**
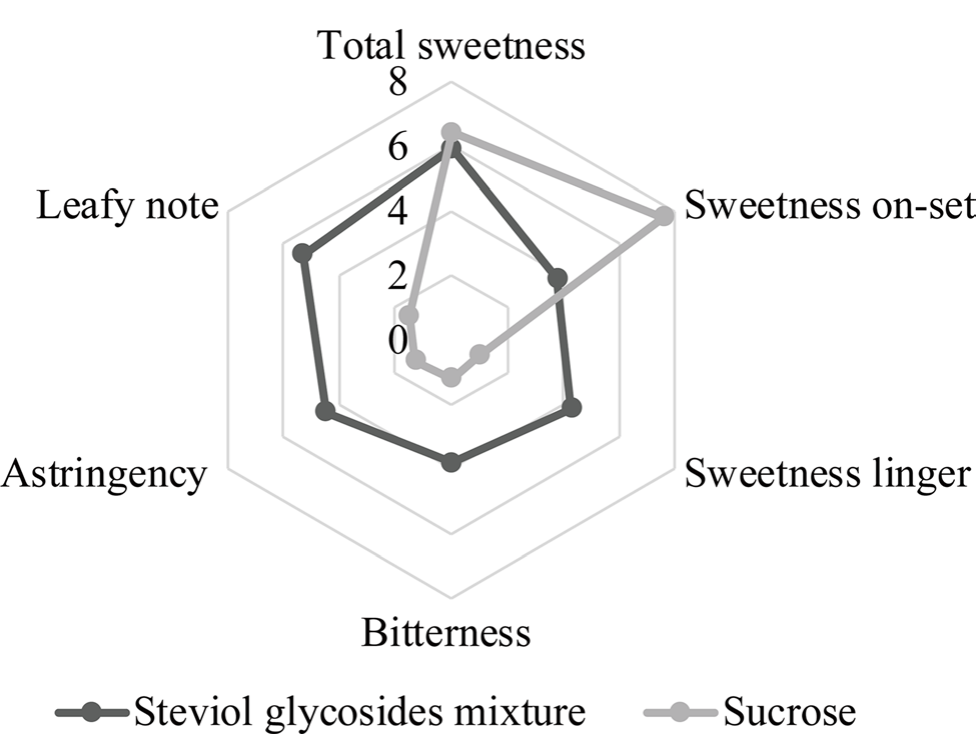
Comparison of sensory attributes of steviol glycosides mixture (0.03%) and sucrose (6%) in iso-sweet water solutions

The SGs mixture used in this study is a white powder provided by Dongtai Hirye Biotechnology Co. Ltd (Dongtai, China) with the product batch number M20220316. The compositions of SGs in the mixture were analyzed using HPLC following China National Standard GB 8270 − 2014, and detailed ingredients are provided in Table 1. The main components of the SGs mixture used in this study are Rebaudioside A (39.90%), Stevioside (30.40%), Rebaudioside C (12.40%), Rebaudioside F (2.00%), Rebaudioside D (1.00%), Rubusoside (0.70%), Rebaudioside B (0.60%), Dulcoside A (0.3%), and Steviolbioside (0.2%), and the total SGs is 87.50%. Sensory evaluation was conducted to assess the characteristics of the SGs mixture [22]. As depicted in Fig. 1, the SGs mixture exhibited a delayed onset of sweetness, with noticeable bitterness, a slight stringency, and an unpleasant metallic aftertaste when compared with sucrose in an iso-sweet water solution.

**Table 1:** Identified components and their contents in the steviol glycosides mixture used in this study (air-dry basis)

Components	Content, %
Stevioside	30.40
Rebaudioside A	39.90
Rebaudioside B	0.60
Rebaudioside C	12.40
Rebaudioside D	1.00
Rebaudioside F	2.00
Dulcoside A	0.30
Rubusoside	0.70
Steviolbioside	0.20
Moisture	3.70
Ash	0.06

High-performance liquid chromatography (HPLC) was used for the identification and quantification of stevioside, rebaudioside A, rebaudioside B, rebaudioside C, rebaudioside D, rebaudioside F, dulcoside A, rubusoside, and steviolbioside following China National Standard GB 8270 − 2014. The contents of moisture and ash were determined according to the China National Standard; and the document numbers are GB/T 6435 − 2014, and GB/T 6438 − 2007, respectively

### Animal management and experimental design

A total of 216 weaned piglets (Duroc × Landrace × Yorkshire) aged 21 days with an average initial body weight (BW) of 7.36 ± 0.04 kg were randomly allocated to six treatments. Each treatment consisted of six replicates, with six pigs per replicate pen, including three barrows and three gilts. The experimental groups comprised a control group receiving a basal diet devoid of any sweeteners, and experimental groups received a basal diet supplemented with 100, 150, 200, 250, and 300 mg/kg SGs mixture (SGs100, SGs150, SGs200, SGs250, and SGs300), respectively. Before the commencement of this trial, the pigs underwent a 5-day acclimation period. The experiment lasted for 42 days. The basal diet (Table 2) was formulated according to the nutrient requirement recommendations for piglets by the NRC (2012). All diets were provided as pellet. Feed intake was recorded on a weekly basis in pen units to collectively measure consumption levels, while individual pig body weights were measured on day 29 and 43. These data facilitated the calculation of the average daily gain (ADG), average daily feed intake (ADFI), and feed to gain ratio (F/G) over the periods of 1 ~ 28 days, 29 ~ 42 days, and 1 ~ 42 days. All piglets were housed individually in pens within a temperature-controlled and enclosed nursery. The pens, measuring 1.2 × 2.1 m^2^, featured high-rise beds equipped with plastic slatted floors and an effective mechanical ventilation system. Each pen was outfitted with two stainless steel feeders and four nipple drinkers, ensuring that all pigs had *ad libitum* access to both feed and water.

**Table 2:** Composition and nutrient levels of the basal diet (air-dry basis)

Ingredients	%	Nutrient levels ^2)^	
Corn	30.34	Digestive energy, MJ/kg	14.69
Puffed corn	20.00	Metabolic energy, MJ/kg	13.55
Puffed soybean	7.02	Net energy, MJ/kg	10.23
Fermented soybean meal	12.00	Crude protein, %	19.78
Soybean meal	6.52	Crude fat, %	4.39
Fish meal	3.20	Ca, %	0.73
Low-protein whey powders	15.00	TP, %	0.51
Soybean oil	1.00	STTD P, %	0.38
Limestone	1.15	SID Lys, %	1.45
CaHPO_4_	0.65	SID Met + Cys, %	0.71
NaCl	0.25	SID Thr, %	0.84
*L*-Lysine hydrochloride	0.78	SID Trp, %	0.21
*DL*-Methionine	0.21	SID Val, %	0.73
*L*-Threonine	0.29		
*L-*Tryptophan	0.03		
Vitamin-mineral premix^1)^	1.56		
Total	100		

1)The premix provided the following per kg of diets: VA 12 400 U, VD_3_ 2 800 U, VE 30 mg, VK_3_ 5 mg, VB_12_ 40 µg, VB_1_ 3 mg, VB_2_ 10 mg, nicotinic acid 40 mg, D-pantothenic acid 15 mg, folic acid 1 mg, VB_6_ 8 mg, biotin 0.08 mg, FeSO_4_•H_2_O 120 mg, CuSO_4_•5H_2_O 16 mg, MnSO_4_•H_2_O 7 mg, ZnSO_4_•H_2_O 80 mg, CaI_2_O_6_ 0.7 mg, Na_2_SeO_3_ 0.30 mg

2)Nutrient levels were calculated values, except that the crude protein, crude fat, Ca, and TP levels were determined according to the China National Standard; and the document numbers are GB/T 6432 − 2018, GB/T 6433 − 2006, GB/T 6436 − 2002 and GB/T 6437 − 2018, respectively

### Sample collection

At the end of the experiment, all the pigs were weighed individually after 16-hours fasting. One pig from each pen (36 pigs total) was randomly selected for blood collection and sacrifice. A 10 mL blood sample was obtained from each pig by anterior vena cava puncture, and serum samples were separated by centrifugation at 1509.3 × *g* at 4 °C for 10 min. Then the pig was anesthetized by intravenous injection of pentobarbital sodium (30 mg/kg BW) and killed by bloodletting. The intestinal tissues were dissected on ice and categorized into the duodenum, jejunum, and ileum. The middle jejunum tissue was sampled, opened, and thoroughly washed with cold sterile phosphate-buffered saline. Mucosa samples were scraped from the inner side of the jejunum using a sterile slide, and then were collected. Approximately 0.5 g of the middle section of *longissimus thoracis* and the central portion of liver tissues from the same region were obtained to assess antioxidant capacity indicators. All samples were immediately transferred to liquid nitrogen for rapid freezing and subsequently stored at -80 °C for further analysis.

### Appetite-associated hormones detection

Appetite-associated hormones such as ghrelin (GHRL), glucagon-like peptide-1 (GLP-1), cholecystokinin (CCK), leptin (LEP), and insulin (INS) in serum were detected by commercial Elisa kits purchased from Jiangsu Meimian Industrial Co., Ltd (Jiangsu, China).

### Real-time qPCR

The jejunal mucosal tissues were processed to extract total RNA using TRIzol reagent (Invitrogen, Carlsbad, CA, USA). The concentration and purity of the RNA were evaluated using a NanoDrop ND-1000 Spectrophotometer (Nano-Drop Technologies, Rockland, DE), followed by monitoring RNA integrity on 1% agarose gels. Subsequently, the first-strand cDNA synthesis was conducted through reverse transcription of 1 µg total RNA utilizing the Prime Script RT reagent kit with gDNA Eraser (Takara, Tokyo, Japan). Real-time qPCR was conducted using the Bio-Rad CFX System in a final volume of 20 µL, comprising 2 µL of cDNA product (diluted at 1:9, v/v), 10 µL of iTaq Universal SYBR Green PCR Supermix (2×, Bio-Rad, Hercules, California, USA), 6.4 µL of RNase-free water, and 0.8 µL of each forward and reverse primers (10 µM/L) as detailed in Table 3. The PCR cycling conditions included an initial denaturation step (95 °C for 30 s) followed by 40 cycles of amplification and quantification (95 °C for 15 s, 60 °C for 30 s, and 72 °C for 30 s with a single fluorescence reading). All measurements were carried out in triplicate. The 2^−ΔΔCT^ method was employed to analyze gene expression levels. *β*-actin was served as the housekeeping gene, and the data were normalized to the control group.

**Table 3:** Primers used for real-time PCR in this study

Items	Nucleotide sequence of primers (5’-3’)	Product size, bp	GenBank Accession	Annealing temperature, °C
*SGLT-1* *(SLC5A)*	Forward: TCATCATCGTCCTGGTCGTCTC Reverse: CTTCTGGGGCTTCTTGAATGTC	144	XM_021072101.1	58
*GLUT2* *(SLC2A2)*	Forward: GCACATCCTGCTTGGTCTATCT Reverse: CACTTGATGCTTCTTCCCTTTC	203	NM_001097417.1	60.5
*GLUT4* *(SLC2A4)*	Forward: AGGCACCCTCACTACCCTCT Reverse: CTTCTTCCTTCCCAGCCACT	109	NM_001128433.1	60
*T1R2*	Forward: TCGCCTCGTGCTGTCATAG Reverse: CCACATCTCAGAGCCTGACC	317	XM_021093444.1	60
*T1R3*	Forward: TGTACCAGGTTCTCGTCCCT Reverse: GGCCATGAACACTAGGCTG	172	NM_001113288.1	60
*β-actin*	Forward: CATCGTCCACCGCAAAT Reverse: TGTCACCTTCACCGTTCC	210	NC_010445	60

Abbreviations: *SGLT-1*, sodium glucose cotransporter-1; *GLUT2*, glucose transporters 2; *GLUT4*, glucose transporters 4; *T1R2*, taste receptor family 1 member 2; *T1R3*, taste receptor family 1 member 3

### Serum biochemical parameters measurement

Serum biochemical parameters including serum glucose (GLU), total protein (TP), albumin (ALB), urea (URE), triglyceride (TG), total cholesterol (CHO), high-density lipoprotein cholesterol (HDL-C), low-density lipoprotein cholesterol (LDL-C), alanine aminotransferase (ALT), aspartate aminotransferase (AST), total bilirubin (TBIL), alkaline phosphatase (ALP), and creatinine (CRE) were detected by commercial kits purchased from Biosino Biotechnology and Science inc. (Beijing, China) with an automatic biochemical analyzer (Selectra Pro XL, EliTechGroup, Puteaux, France).

### Serum immunoglobulins, cytokine determination

Serum immunoglobulin A (IgA), IgG, and IgM, and cytokines interleukin-1 (IL-1), IL-1*β*, IL-6, IL-8, IL-10, IL-22, interferon-γ (IFN-*γ*), tumor necrosis factor-α (TNF-*α*), and TNF-*β* were measured using commercial kits obtained from Jiangsu Meimian Industrial Co., Ltd (Jiangsu, China).

### Antioxidant capacity evaluation

Malondialdehyde (MDA), total antioxidant capacity (T-AOC), total superoxide dismutase (T-SOD), catalase (CAT), and glutathione peroxidase (GSH-Px) were assessed using commercial kits purchased from Nanjing Jiancheng Bioengineering Institute (Nanjing, China). Samples of liver and *longissimus thoracis* tissues were homogenized in physiological saline, and the supernatants were harvested after centrifugation for subsequent analysis. The final data were normalized to the total protein concentration in the tissues.

### Statistical analysis

The data were analyzed using IBM SPSS Statistics V18.0 software (IBM Corp., Armonk, NY, USA). Results were presented as means with a pooled standard error (SEM). Prior to intergroup difference analysis, the normality of the data was assessed using the Shapiro-Wilk test. For variables with non-normal distributions, analysis was performed using one-way ANOVA followed by the Kruskal-Wallis test with false discovery rate (FDR) multiple corrections. When the data exhibited a normal distribution, one-way ANOVA analysis followed by the LSD test was employed. The linear and quadratic responses of parameters to different SGs mixture supplemental levels were evaluated using regression analysis with a curve estimation model. Each pen was considered as the experimental unit for the data of growth performance, while individual pigs served as the experimental unit for the other data. Statistical significance was considered at *P* < 0.05, with a significant trend noted at 0.05 ≤ *P* < 0.10.

## Results

### Growth performance

As illustrated in Table 4, increasing SGs mixture supplemental level from 0 to 300 mg/kg linearly (*P* < 0.05) and quadratically (*P* < 0.10) decreased ADG from days 29 to 42, as well as ADG from days 1 to 42 (*P* < 0.10). Furthermore, the ADFI from days 1 to 42 showed a trend of initially increasing, followed by a decrease as the level of dietary SGs mixture supplementation rose (*P* < 0.10), with the highest ADFI recorded in the group supplemented with 100 mg/kg of the SGs mixture. However, inclusion the SGs mixture in the diet did not have a significant impact on the ADG, ADFI, or F/G from days 1 to 28 in the piglets (*P* > 0.05).

**Table 4:** Effects of dietary steviol glycosides mixture supplementation on growth performance of weaned piglets

Items	Steviol glycosides mixture, mg/kg						SEM	*P*-value		
	0	100	150	200	250	300		ANOVA	Linear	Quadratic
Day 1 ~ 28										
ADG, g/d	370	380	400	369	370	367	4.22	0.216	0.160	0.221
ADFI, g/d	554	534	562	538	542	535	5.78	0.485	0.248	0.514
F/G	1.47	1.41	1.41	1.45	1.47	1.46	0.01	0.134	0.696	0.268
Day 29 ~ 42										
ADG, g/d	613	615	586	583	582	572	6.90	0.280	0.030	0.081
ADFI, g/d	972	979	991	934	955	939	9.01	0.430	0.111	0.219
F/G	1.60	1.67	1.67	1.60	1.69	1.66	0.01	0.159	0.179	0.354
Day 1 ~ 42										
ADG, g/d	430	436	431	415	420	410	3.97	0.371	0.052	0.098
ADFI, g/d	668	694	679	676	671	647	5.63	0.115	0.133	0.060
F/G	1.55	1.59	1.58	1.63	1.60	1.58	0.01	0.942	0.314	0.374

Values are mean and pooled SEM, *n* = 6

Data in the same row with no or the same letter indicate no significant difference (*P*>0.05), while with different letters mean significant difference (*P*<0.05)

Abbreviations: BW, body weight; ADG, average daily gain; ADFI, average daily feed intake; F/G, feed to gain ratio

The *P* values indicate the effects of dietary steviol glycosides mixture supplementation with different levels by one-way ANOVA and linear and quadratic analyses, respectively

### Appetite-associated hormones

As displayed in Table 5, increasing SGs mixture supplementation resulted in a linear increase in the serum CCK content of weaned piglets (*P* < 0.05). However, there were no significant differences in serum GHRL, GLP-1, LEP, and INS levels among the different treatments (*P* > 0.05).

**Table 5:** Effects of dietary steviol glycosides mixture supplementation on serum appetite-related hormones in weaned piglets

Items	Steviol glycosides mixture, mg/kg						SEM	*P*-value		
	0	100	150	200	250	300		ANOVA	Linear	Quadratic
GHRL, ng/L	1465.82	1547.11	1567.44	1584.63	1403.29	1528.35	26.78	0.371	0.999	0.527
GLP-1, pM/L	2.36	2.13	1.95	2.23	2.27	2.42	0.07	0.370	0.630	0.105
CCK, ng/L	468.22^c^	491.70^c^	527.17^bc^	531.87^abc^	586.64^ab^	620.03^a^	14.64	0.015	< 0.001	0.001
LEP, ng/L	4062.98	3574.79	3915.89	3829.74	4180.65	3630.82	76.25	0.140	0.688	0.864
INS, mU/L	68.53	59.73	67.83	62.28	61.58	58.51	1.31	0.115	0.077	0.213

Values are mean and pooled SEM, *n* = 6

Data in the same row with no or the same letter indicate no significant difference (*P*>0.05), while with different letters mean significant difference (*P*<0.05)

Abbreviations: GHRL, ghrelin; GLP-1, glucagon-like peptide-1; CCK, cholecystokinin; LEP, leptin; INS, insulin

The *P* values indicate the effects of dietary steviol glycosides mixture supplementation with different levels by one-way ANOVA and linear and quadratic analyses, respectively

^abc^Means in the same row with different superscripts differ (*P* < 0.05)

### **Gene expression of gut chemoreceptors**

**Fig. 2:**
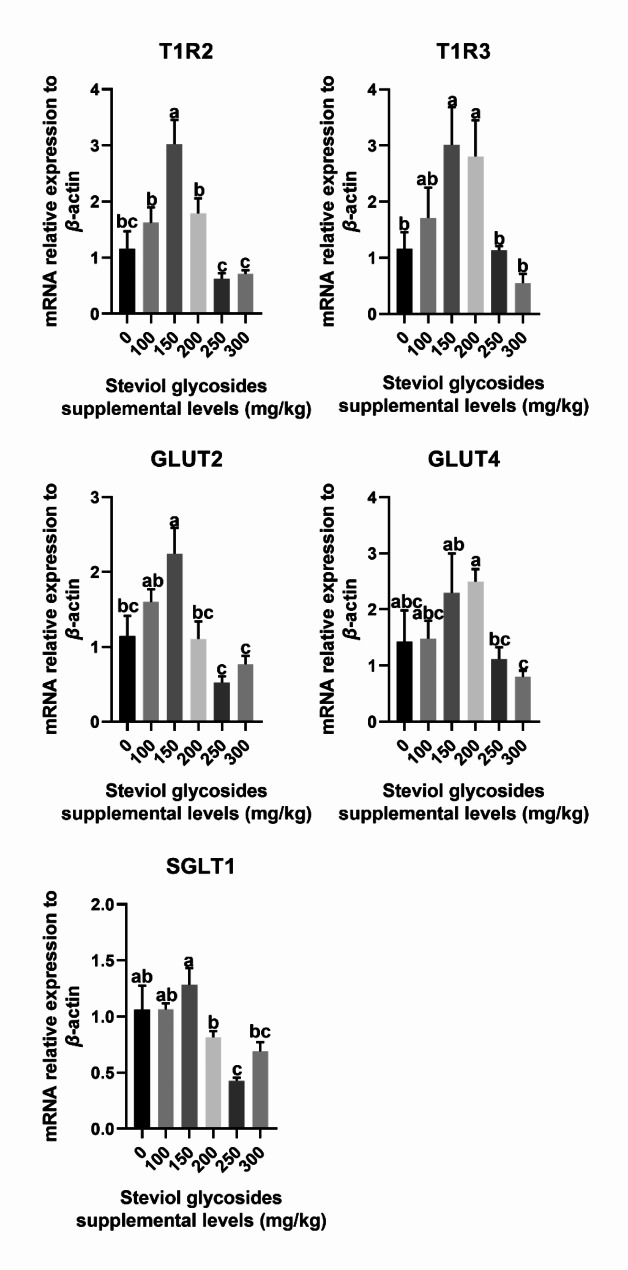
Bar graphs show the effect of dietary steviol glycosides mixture supplementation on the relative mRNA expression of chemoreceptors in the jejunum of weaned piglets. All data are expressed as the mean ± SEM (*n* = 6). Differences were determined by one-way ANOVA followed by LSD test. ^abc^ Means in the columns with different superscripts differ (*P* < 0.05)

As shown in Figs. 2 and 150 mg/kg SGs mixture supplementation resulted in a significant up-regulation of the relative mRNA expression of *T1R2* and *GLUT2* in the jejunal mucosa compared with the control group (*P* < 0.05). In addition, the relative mRNA expression of *T1R3* in the jejunal mucosa was significantly increased by 150 and 200 mg/kg SGs mixture supplementation (*P* < 0.05). Conversely, 250 mg/kg SGs mixture supplementation significantly decreased the mRNA expression of the jejunal mucosa *SGLT1* gene compared with the control group (*P* < 0.05). However, the addition of SGs mixture did not affect the mRNA expression of the jejunal mucosa *GLUT4* gene in weaned piglets (*P* > 0.05).

### Biochemical parameters

As shown in Table 6, increasing SGs mixture supplementation linearly decreased serum GLU levels (*P* < 0.05). Furthermore, 100 and 150 mg/kg SGs mixture supplementation significantly reduced the concentration of serum TBIL compared with the control group (*P* < 0.05). However, the SGs mixture supplementation did not have a significant impact on the levels of serum TP, ALB, URE, TG, CHO, HDL-C, LDL-C, ALT, AST, ALP, and CRE (*P* > 0.05).

**Table 6:** Effects of dietary steviol glycosides mixture supplementation on serum biochemical parameters of weaned piglets

Items	Steviol glycosides mixture, mg/kg						SEM	*P*-value		
	0	100	150	200	250	300		ANOVA	Linear	Quadratic
GLU, mM/L	2.58	1.89	1.99	1.95	1.83	1.97	0.09	0.116	0.028	0.017
TP, g/L	61.19	59.02	59.80	57.33	55.20	59.71	0.89	0.462	0.215	0.336
ALB, g/L	37.52	37.32	32.53	33.37	38.94	37.11	0.99	0.358	0.971	0.388
URE, mM/L	5.14	4.27	4.11	4.51	4.79	4.48	0.19	0.700	0.577	0.404
TG, mM/L	0.52	0.54	0.47	0.51	0.54	0.43	0.02	0.640	0.446	0.639
CHO, mM/L	9.92	10.22	10.71	10.73	10.69	10.14	0.21	0.829	0.496	0.423
HDL-C, mM/L	0.89	0.83	0.85	0.83	0.82	0.84	0.02	0.889	0.316	0.487
LDL-C, mM/L	1.47	1.41	1.44	1.41	1.35	1.41	0.04	0.970	0.476	0.770
ALT, U/L	51.28	44.10	43.37	50.06	44.86	42.23	1.60	0.491	0.221	0.454
AST, U/L	50.93	55.35	50.43	56.98	62.95	50.70	2.76	0.778	0.594	0.775
TBIL, µM/L	16.50^a^	10.45^b^	10.07^b^	14.32^ab^	15.65^a^	14.66^ab^	0.74	0.034	0.785	0.039
ALP, U/L	250.98	199.39	208.73	237.89	237.96	202.52	7.62	0.226	0.402	0.556
CRE, µM/L	105.87	101.45	95.94	101.12	98.51	102.46	1.42	0.464	0.369	0.194

Values are mean and pooled SEM, *n* = 6

Data in the same row with no or the same letter indicate no significant difference (*P*>0.05), while with different letters mean significant difference (*P*<0.05)

Abbreviations: GLU, glucose; TP, total protein; ALB, albumin; URE, urea; TG, triglyceride; CHO, cholesterol; HDL-C, high-density lipoprotein cholesterol; LDL-C, low-density lipoprotein cholesterol; ALT, alanine aminotransferase; AST, aspartate aminotransferase; TBIL, total bilirubin; ALP, alkaline phosphatase; CRE, creatinine

The *P* values indicate the effects of dietary steviol glycosides mixture supplementation with different levels by one-way ANOVA and linear and quadratic analyses, respectively

^abc^Means in the same row with different superscripts differ (*P* < 0.05)

### Immunological function indicators

As shown in Table 7, increasing SGs mixture supplementation linearly decreased the proinflammatory cytokine IL-1β levels in serum (*P* < 0.05). However, there were no significant differences in the levels of serum IgA, IgG, IgM, IL-1, IL-6, IL-8, TNF-α, TNF-β, IL-10, and IL-22 among different treatments (*P* > 0.05).

**Table 7:** Effects of dietary steviol glycosides mixture supplementation on immunological function indicators of weaned piglets

Items	Steviol glycosides mixture, mg/kg						SEM	*P*-value		
	0	100	150	200	250	300		ANOVA	Linear	Quadratic
IgA, µg/mL	17.12	15.35	15.78	15.70	15.91	17.34	0.48	0.809	0.943	0.339
IgG, µg/mL	293.70	352.85	320.66	346.67	331.16	303.40	7.53	0.161	0.869	0.071
IgM, µg/mL	22.72	25.70	23.84	22.81	23.52	26.14	0.48	0.152	0.308	0.586
IL-1, ng/L	74.66	73.42	71.57	72.15	75.19	77.84	1.32	0.812	0.499	0.346
IL-1β, ng/L	30.29	28.21	22.06	26.02	24.15	21.20	1.19	0.189	0.022	0.072
IL-6, ng/L	833.32	764.22	810.74	768.28	788.15	794.03	13.78	0.720	0.349	0.457
IL-8, ng/L	292.64	311.34	299.70	292.43	328.00	336.04	6.45	0.259	0.058	0.116
TNF-α, pg/mL	265.14	258.95	221.31	272.57	246.57	246.57	6.82	0.348	0.566	0.685
TNF-β, ng/L	185.94	157.97	149.56	138.00	184.05	174.58	8.43	0.506	0.875	0.250
IL-10, ng/L	66.77	69.34	68.97	70.44	68.97	67.75	1.99	0.997	0.849	0.869
IL-22, ng/L	17.68	15.23	15.95	15.00	17.86	14.60	0.59	0.485	0.412	0.633

Values are mean and pooled SEM, *n* = 6

Data in the same row with no or the same letter indicate no significant difference (*P*>0.05), while with different letters mean significant difference (*P*<0.05)

Abbreviations: IgA, immunoglobulin A; IgG immunoglobulin G; IgM immunoglobulin M; IL-1, interleukin-1; IL-1β, interleukin-1β; IL-6, interleukin-6; IL-8, interleukin-8; IFN-γ, interferon-γ; TNF-α, tumor necrosis factor-α; TNF-β, tumor necrosis factor-β; IL-10, interleukin-10; IL-22, interleukin-22

The *P* values indicate the effects of dietary steviol glycosides mixture supplementation with different levels by one-way ANOVA and linear and quadratic analyses, respectively

### Antioxidant capacity

As shown in Tables 8 and 150 mg/kg SGs mixture supplementation resulted in a significant reduction in serum MDA content compared with the control group (*P* < 0.05). Increasing SGs mixture supplementation from 0 to 300 mg/kg linearly and quadratically increased serum T-SOD, CAT, and GSH-Px activities (*P* < 0.05). Piglets fed a diet supplemented with 100 mg/kg SGs mixture had higher serum T-SOD, CAT, and GSH-Px activities (*P* < 0.05). Moreover, increasing SGs mixture supplementation linearly increased hepatic T-AOC content (*P* < 0.05). Furthermore, increasing SGs mixture supplementation linearly and quadratically increased the hepatic T-SOD and GSH-Px activity, as well as muscle T-AOC content (*P* < 0.05). In contrast, increasing SGs mixture supplementation linearly and quadratically decreased the MDA content and T-SOD activity in the muscle (*P* < 0.05).

**Table 8:** Effects of dietary steviol glycosides mixture supplementation on the antioxidant capacity of weaned piglets

Items	Steviol glycosides mixture, mg/kg						SEM	*P*-value		
	0	100	150	200	250	300		ANOVA	Linear	Quadratic
Serum										
MDA, nM/mL	3.92^a^	3.03^ab^	2.13^b^	4.06^a^	3.59^a^	3.31^ab^	0.19	0.022	0.862	0.331
T-AOC, mM/L	1.01	0.94	0.98	0.94	0.93	1.00	0.01	0.251	0.559	0.142
T-SOD, U/mL	193.13^b^	221.53^a^	225.08^a^	170.61^c^	147.43^d^	168.06^cd^	5.60	< 0.001	0.001	< 0.001
CAT, U/mL	1.66^b^	7.27^a^	6.98^a^	6.96^a^	6.10^a^	6.08^a^	0.38	< 0.001	0.001	< 0.001
GSH-Px, U/L	782.47^b^	1089.32^a^	1065.21^a^	1019.18^a^	1003.84^a^	1044.38^a^	21.02	< 0.001	0.002	< 0.001
Liver										
MDA, nM/mg prot	0.33	0.27	0.19	0.27	0.25	0.22	0.02	0.489	0.120	0.217
T-AOC, mM/g prot	0.08	0.10	0.12	0.13	0.12	0.12	0.01	0.392	0.030	0.069
T-SOD, U/mg prot	545.72^c^	591.17^bc^	600.63^bc^	714.36^abc^	742.36^ab^	795.00^a^	27.76	0.047	0.001	0.004
CAT, U/mg prot	44.05	49.78	52.46	47.24	51.66	45.39	1.72	0.691	0.696	0.358
GSH-Px, U/mg prot	8.25^b^	33.41^a^	28.73^a^	30.44^a^	34.58^a^	30.96^a^	2.13	0.001	0.002	< 0.001
*Longissimus thoracis*										
MDA, nM/mg prot	0.15^a^	0.11^b^	0.09^b^	0.09^b^	0.09^b^	0.08^b^	0.01	0.010	< 0.001	0.001
T-AOC, mM/g prot	0.02^a^	0.03^a^	0.02^ab^	0.02^bc^	0.02^c^	0.02^bc^	0.00	< 0.001	0.001	0.003
T-SOD, U/mg prot	73.86^a^	76.52^a^	62.68^b^	65.83^b^	61.13^b^	61.36^b^	1.41	0.001	< 0.001	0.001
CAT, U/mg prot	0.35	0.44	0.40	0.47	0.48	0.30	0.02	0.104	0.466	0.072
GSH-Px, U/mg prot	2.32	3.99	3.36	2.16	3.03	2.85	0.29	0.447	0.741	0.763

Values are mean and pooled SEM, *n* = 6

Data in the same row with no or the same letter indicate no significant difference (*P*>0.05), while with different letters mean significant difference (*P*<0.05)

Abbreviations: MDA, malondialdehyde; T-AOC total antioxidant capacity; T-SOD, total superoxide dismutase; CAT, catalase; GSH-px, glutathione peroxidase; prot, protein

The *P* values indicate the effects of dietary steviol glycosides mixture supplementation with different levels by one-way ANOVA and linear and quadratic analyses, respectively

^abc^Means in the same row with different superscripts differ (*P* < 0.05)

## Discussion

The utilization of SGs in livestock and poultry production has attracted significant attention due to their beneficial effects on enhancing production performance, feed efficiency, and the quality of animal products. For instance, research conducted by Jiang et al. [14] demonstrated that dietary 250 mg/kg stevioside supplementation significantly increased body weight, ADG, and ADFI in Ross 308 broiler chickens. Furthermore, another study revealed that including 80 mg/kg of stevia-based sweeteners (which contained 0.5% SGs) in the diets of Cobalt line broiler chickens for 42 days significantly enhanced final body weight and ADG [23]. However, it is worth noting that Wu et al. [24] reported no significant effect of stevioside on ADG, ADFI, F/G, and immune organ index in Arbor Acres broilers. In a study involving Shandong black goats, Han et al. [16] reported that adding 400 to 800 mg/kg of stevioside significantly increased both forage hay consumption and total feed intake. Furthermore, introducing 0.3% stevioside into the diet of Hanwoo cattle enhanced final weight, weight gain, and carcass crude protein content, while also reducing drip loss, shear force, and increasing meat color redness value in *longissimus thoracis*, thereby improving meat quality [25]. Additionally, including 0.3% stevia in the diet of growing pigs significantly improved ADG, feed utilization, body immunity, and carcass traits including backfat thickness [26]. A study indicated that increasing the concentration of stevioside or rebaudioside A from 0 to 300 mg/kg led to a linear increase in ADFI and ADG, while also resulting in a linear decrease in F/G from days 1 to 28 in weaned piglets [27]. Moreover, the addition of 167 mg/kg of stevia significantly boosted the ADG of weaned piglets in the second week post-weaning. However, varying proportions of stevia (0.0833%, 0.167%, or 0.334%) did not significantly affect feed intake or F/G in weaned piglets [18], which is consistent with similar findings from this experiment noting no significant effect of the SGs mixture on ADG, ADFI, and F/G in weaned piglets. Our research revealed a linear and quadratic relationship between the ADG and the SGs mixture supplemental levels, with ADFI exhibiting an initial increase followed by a decrease as the amount of SGs mixture in the diet increased. This implies that the effectiveness of SGs does not necessarily conform to a “more is better” principle.

The combination of certain sweeteners often leads to a synergistic sweetness effect [28]. A study conducted by Tian et al. [22] to assess the temporal perception of sweetness and bitterness for six commonly steviol glycosides indicated that Rubusoside and Stevioside display an immediate and pronounced bitter taste with a lingering aftertaste. In addition, SGs are commonly associated with a somewhat unpleasant bitter aftertaste, particularly at high concentration [29]. The main components of the SGs mixture used in this study are Rebaudioside A (39.90%) and Stevioside (30.40%). Therefore, the SGs mixture may show an unfavorable aftertaste experience for the piglets due to these reasons, despite the sweetness.

Sweet taste receptor cells were regulated by at least two signaling pathways, one mediated by a heterodimeric G-protein coupled receptor encoded by T1R2/T1R3 genes and another by glucose transporters and the ATP-gated potassium (K_ATP_) channel [30]. The perception of sweetness is mainly facilitated by the sweet taste receptor T1R2/T1R3 present in taste cells of the lingual epithelium [31]. However, sweet taste receptors are also found in intestinal enteroendocrine cells [19, 32, 33]. Non-nutritive sweeteners have been shown to stimulate the mRNA expression of T1R2/T1R3 in the intestine of pigs [34]. Stevioside can enhance the function of the sweet taste transduction receptor called transient receptor potential melastatin 5 (TRPM5) [35], which is a calcium-activated cation channel present in type II taste receptor cells. In humans, stevioside was also reported to activate mRNA expression of T1R2/T1R3 [36]. Accordingly, our findings suggest that dietary SGs mixture supplementation can activate the mRNA expression of T1R2 and T1R3 in the jejunal mucosa of piglets.

A 2-dimensional organoid intestinal model experiment indicated that Rebaudioside A can significantly induce GLP-1 and CCK secretion [37]. GLP-1 is secreted from L cells in the intestine and exerts a strong incretin effect by enhancing insulin secretion in response to glucose levels and slowing down gastric emptying and motility [38]. Experiments with mouse and human intestinal enteroendocrine cell lines confirmed that Rebaudioside A stimulated GLP-1 release in a concentration-dependent manner via bitter taste signaling pathways. Contrary to this, we did not observe a rising in serum GLP-1 levels with increasing dietary SGs mixture supplementation. The discrepancy could be attributed to the short half-life of GLP-1 in the bloodstream. The levels of GLP-1 in plasma may not be a precise indicator of its local release in the intestine [39]. Consistently, we found a linear increase in serum CCK levels with increasing of SGs supplementation. CCK is released postprandially from the I cell of the small intestine into the bloodstream, and it has been reported to reduce food intake in both humans and rodents [40]. In the present study, the ADFI from days 1 to 42 decreased when the dietary supplementation of the SGs mixture reached 300 mg/kg. Our data suggest that the SGs mixture may function as an appetite suppressant when supplemented at a high concentration.

SGs are considered as a promising phytomedicine for managing diabetes. As a low-calorie, intensely sweet sugar substitute, it plays a pivotal role in improving the body’s blood glucose profile. Existing studies have indicated the effectiveness of stevia consumption in reducing postprandial blood glucose and insulin levels compared with aspartame and sucrose across lean and obese subjects [41]. When orally administered, SGs are resistant to degradation by gastric acid and digestive enzymes in the digestive tract. Only a small portion of SGs can be completely broken down into the aglycone steviol and glucose by the intestinal microflora in the lower intestinal tract [42–44]. Despite its sweetness, the limited absorption of SGs prevents a rapid spike in blood glucose levels post-ingestion. Deenadayalan et al. [45]. demonstrated that stevioside can effectively promote glucose uptake in diabetic gastrocnemius muscles by activating the insulin receptor (IR)/insulin receptor substrate-1(IRS-1)/Akt/GLUT4 pathway. Another study has suggested that SGs can mimic the effects of insulin by influencing GLUT translocation through the phosphatidylinositol 3-kinase (PI3K)/protein kinase B (Akt) pathway [46]. Interestingly, we observed an upregulation of GLUT2 mRNA expression in the jejunum and a linear decrease in serum GLU content, but the underlying mechanism needs further research.

In broiler chickens, dietary SGs supplementation has been linked to elevated antibody levels against the Newcastle disease virus [23]. Insights into the mechanism of action of steviol glycosides have unveiled their modulatory effects on immune responses. These effects include the attenuation of pro-inflammatory factors like TNF-α, IL-1β, and IL-6 in mastitis-modeled mice by suppressing the toll-like receptor 2 (TLR2), nuclear factor kappa B (NF-κB), and MAPK pathways [47]. A study has indicated that SGs and steviol may have the potential to inhibit the release of pro-inflammatory cytokines such as TNF-α, IL-1β, and IL-6 induced by lipopolysaccharides (LPS) by modulating cytokine gene expression through the Iκ-Bα/NF-κB signaling pathway [48]. Consistent with these findings, our result demonstrates a significant linear decrease in serum IL-1β level with increasing SGs mixture supplementation, and indicates the potential of enhancing immune function and reducing inflammation of SGs mixture.

Weaning stress can damage the oxidation-antioxidant system and induce oxidative stress by decreasing the activity of SOD and increasing the concentration of MDA and NO [49]. Stevioside has been shown to mitigate diquat-induced cytotoxicity, inflammation, and apoptosis in IPEC-J2 cells, preserving cellular barrier integrity and combating oxidative stress by modulating the NF-κB and mitogen-activated protein kinase (MAPK) signaling pathways [21]. In diabetic rats, SGs treatments have also demonstrated the ability to significantly enhance the activity of T-SOD and CAT in the liver [50]. Moreover, administration of 50 mg/kg stevioside has been reported to decrease oxidative stress markers such as 4-hydroxynonenal (4-HNE) and 3-nitrotyrosine (3-NT) and alleviate cisplatin-induced oxidative stress [51]. Furthermore, dietary stevioside supplementation normalized LPS-induced changes in protein expression of the antioxidant genes of nuclear factor-erythroid 2-related factor 2 (Nrf-2) and heme oxygenase-1 (HO-1), and ameliorated the redox damage by reducing MDA content and increasing total antioxidant capacity in boiler chickens [52]. In aged breeder hens, 0.25 g/kg stevioside supplementation significantly enhanced the antioxidant capacity of the ovary and shell glands by increasing the activity of CAT, SOD, or GSH-Px and reducing the MDA content [53]. Similarly, our findings also highlight that dietary SGs mixture supplementation can improve the antioxidant capacity of weaned piglets.

## Conclusions

In conclusion, our findings indicate that dietary 100 ~ 150 mg/kg SGs mixture supplementation modulates gene expression of sweet taste recognition receptors and glucose transporters, while also enhancing the antioxidant capacity of weaned piglets.

## Data Availability

No datasets were generated or analysed during the current study.

## Abbreviations

SGs: Steviol glycosides
BW: Body weight
ADG: Average daily gain
ADFI: Average daily feed intake
F/G: Feed to gain ratio
GHRL: Ghrelin
GLP-1: Glucagon-like peptide-1
CCK: Cholecystokinin
LEP: Leptin
INS: Insulin
SGLT-1: Sodium glucose cotransporter-1
GLUT2: Glucose transporters 2
GLUT4: Glucose transporters 4
T1R2: Taste receptor family 1 member 2
T1R3: Taste receptor family 1 member 3
GLU: Glucose
TP: Total protein
ALB: Albumin
URE: Urea
TG: Triglyceride
CHO: Total cholesterol
HDL-C: High-density lipoprotein cholesterol
LDL-C: Low-density lipoprotein cholesterol
ALT: Alanine aminotransferase
AST: Aspartate aminotransferase
TBIL: Total bilirubin
ALP: Alkaline phosphatase
CHO: Total cholesterol
CRE: Creatinine
IgA: Immunoglobulin A
IL-1: Interleukin-1
IFN-γ: Interferon-γ
TNF-α: Tumor necrosis factor-α
MDA: Malondialdehyde
T-AOC: Total antioxidant capacity
T-SOD: Total superoxide dismutase
CAT: Catalase
GSH-Px: Glutathione peroxidase
TLR2: Toll-like receptor 2
NF-κB: Nuclear factor kappa B
MAPK: Mitogen-activated protein kinase
LPS: Lipopolysaccharides
4-HNE: 4-hydroxynonenal
3-NT: 3-nitrotyrosine
Nrf-2: Factor-erythroid 2-related factor 2
HO-1: Heme oxygenase-1
